# EDITING CDKN1A AND RASD1 IN A CELL CULTURE SYSTEM

**DOI:** 10.1093/geroni/igad104.3122

**Published:** 2023-12-21

**Authors:** Hope Vue, Kellie Agrimson

**Affiliations:** St. Catherine University, St. Paul, Minnesota, United States; St. Catherine University, St. Paul, Minnesota, United States

## Abstract

As an individual ages, there is a higher incidence of cancer and infertility. Testicular germ cell cancer rates have been rising year after year. According to the American Cancer Society 1 of every 250 males will develop testicular cancer during their lifetime. In this research project, we aim to develop a molecular toolbox to analyze gene function in male germinal cells using a CRISPR/Cas9 cell culture system. This biotechnology allows for the targeted alteration and cutting of specific DNA strands. To achieve this, we will utilize cancer cell lines (SUSA and NT2/D1 testicular cancer cell lines) and knock out two genes of interest: cyclin-dependent kinase inhibitor 1 (CDKN1A) and ras-related dexamethasone-induced 1 (RASD1). Our objective is to investigate the effects of gene knockout on cell proliferation. For this purpose, we designed primers and single-guide RNAs using bioinformatic databases: Benchling and Chop Chop. The tools we used were employed to amplify human genomic DNA and assess the effectiveness of the guide RNAs through in vitro cutting of our genes of interest, followed by gel electrophoresis observations. Based on the results obtained from these experiments, our next step is to electroporate the guide RNA and Cas9 complex to alter gene function in our cell culture system. Through a better understanding of gene function in testicular cancer cell lines, we hope to inform future therapeutic targets for both testicular cancer and age-related infertility.

